# Deep learning with deep convolutional neural network using FDG-PET/CT for malignant pleural mesothelioma diagnosis

**DOI:** 10.18632/oncotarget.27979

**Published:** 2021-06-08

**Authors:** Kazuhiro Kitajima, Hidetoshi Matsuo, Atsushi Kono, Kozo Kuribayashi, Takashi Kijima, Masaki Hashimoto, Seiki Hasegawa, Takamichi Murakami, Koichiro Yamakado

**Affiliations:** ^1^Department of Radiology, Hyogo College of Medicine, Nishinomiya, Hyogo, Japan; ^2^Department of Radiology, Kobe University Graduate School of Medicine, Kobe, Hyogo, Japan; ^3^Division of Respiratory Medicine, Department of Internal Medicine, Hyogo College of Medicine, Nishinomiya, Hyogo, Japan; ^4^Department of Thoracic Surgery, Hyogo College of Medicine, Nishinomiya, Hyogo, Japan

**Keywords:** mesothelioma, artificial intelligence, deep learning, FDG (fluorodeoxyglucose), PET-CT (positron emission tomography-computed tomography)

## Abstract

Objectives: This study analyzed an artificial intelligence (AI) deep learning method with a three-dimensional deep convolutional neural network (3D DCNN) in regard to diagnostic accuracy to differentiate malignant pleural mesothelioma (MPM) from benign pleural disease using FDG-PET/CT results.

Results: For protocol A, the area under the ROC curve (AUC)/sensitivity/specificity/accuracy values were 0.825/77.9% (81/104)/76.4% (55/72)/77.3% (136/176), while those for protocol B were 0.854/80.8% (84/104)/77.8% (56/72)/79.5% (140/176), for protocol C were 0.881/85.6% (89/104)/75.0% (54/72)/81.3% (143/176), and for protocol D were 0.896/88.5% (92/104)/73.6% (53/72)/82.4% (145/176). Protocol D showed significantly better diagnostic performance as compared to A, B, and C in ROC analysis (*p* = 0.031, *p* = 0.0020, *p* = 0.041, respectively).

Materials and Methods: Eight hundred seventy-five consecutive patients with histologically proven or suspected MPM, shown by history, physical examination findings, and chest CT results, who underwent FDG-PET/CT examinations between 2007 and 2017 were investigated in a retrospective manner. There were 525 patients (314 MPM, 211 benign pleural disease) in the deep learning training set, 174 (102 MPM, 72 benign pleural disease) in the validation set, and 176 (104 MPM, 72 benign pleural disease) in the test set. Using AI with PET/CT alone (protocol A), human visual reading (protocol B), a quantitative method that incorporated maximum standardized uptake value (SUVmax) (protocol C), and a combination of PET/CT, SUVmax, gender, and age (protocol D), obtained data were subjected to ROC curve analyses.

Conclusions: Deep learning with 3D DCNN in combination with FDG-PET/CT imaging results as well as clinical features comprise a novel potential tool shows flexibility for differential diagnosis of MPM.

## INTRODUCTION

Malignant pleural mesothelioma (MPM) is a type of cancer induced by asbestos, though difficult to diagnose. Affected patients are known to have a poor prognosis, thus early testing to discriminate between benign and malignant pleural disease is important for effective treatment, as well as extending survival. Traditionally, chest radiography and computed tomography (CT) imaging are used to examine patients with pleural diseases, while histological results are also employed. While cytologic evaluations of results obtained in pleural fluid and needle aspiration pleural biopsy tests show poor sensitivity for MPM diagnosis [1], improved diagnostic accuracy with use of an image-guided core needle biopsy procedures (67% with ultrasound guidance, 82% with CT guidance) has been reported [2]. Should a larger specimen be needed for diagnosis, open needle biopsy, video-assisted thoracoscopic surgery (VATS), and open biopsy [3] are available options. Of those, VATS has been shown to have a diagnostic rate of 98%, though can be performed only when visceral and parietal pleural surfaces do not show adherence, while the chest wall seeding rate is 50% for VATS as compared to 22% for image-guided biopsy examinations [1–3].

For MPM diagnosis, ^18^F-fluorodeoxyglucose positron emission tomography/computed tomography (FDG-PET/CT) findings, which often include unilateral circumferential or near-circumferential pleural and fissural thickening indicating FDG avidity, are generally utilized. In fact, several groups have reported use of visual analysis or semiquantitative measurements (maximum standardized uptake value; SUVmax) to demonstrate the clinical utility of FDG-PET and PET/CT for discriminating MPM from inflammatory conditions and benign pleural tumors, with sensitivity, specificity, accuracy in those reports ranging from 60–100%, 62–100%, and 84–98%, respectively [4–10]. In a meta-analysis of 407 patients with MPM and 232 with benign pleural conditions, FDG-PET/CT findings were used for differentiation of MPM from benign pleural disease, with pooled sensitivity and specificity found to be 81% and 74%, respectively, and an area under the receiver operating characteristic (ROC) curve value of 0.838 [11]. Furthermore, image-guided and surgical biopsy procedures can be planned by using FDG-PET/CT results, as sites with greatest FDG uptake and/or most accessible can be identified, and then targeted for obtaining tissue samples. On the other hand, for cases with sub-centimeter cancers, low-volume MPM, or low-grade MPM variants, FDG-PET imaging has poor sensitivity, as PET/CT cameras currently available have a limited spatial resolution of approximately 5–6 mm [11, 12] and specificity can also be altered. Nevertheless, a variety of inflammatory conditions are revealed by FDG uptake, including pleuritis, chronic granulomatous inflammation, benign asbestosis plaque, parapneumonic effusion, and talc pleurodesis, as well as some benign mass types, such as solitary fibrous tumor.

In recent years, A deep learning method that utilizes a deep convolutional neural network (DCNN) has received attention for image pattern recognition and artificial intelligence (AI) strategies. Neural networks are based on brain structure and function, and can be utilized for deep machine learning. Mimicking of the visual cortex in mammals can be done when processing data by use of an artificial neural network that contains hidden layers as well as a convolution layer, in which several types of filters are used to process images, and has been shown to be effective for image pattern recognition [13, 14]. While conventional machine learning algorithms require features extracted from images prior to learning, deep learning in contrast can extract meaningful features from images, and then compute inferences and decisions in an autonomous manner. Recent studies have shown that the performance of DCNN-based AI matched or exceeded the capabilities of trained experts in a variety of different medical fields [15, 16]. Thus, it is considered that this learning method has potential to provide diagnosis based on imaging without the need for an experienced radiologist.

DCNNs are generally used with two-dimensional (2D) images in both medical and non-medical settings, whereas reports of applications for three-dimensional (3D) structures, e.g., segmentation of brain lesions, are limited [17]. In the present study, the usefulness of 3D DCNN was examined by extending a network typically used for 2D DCNN with a deep learning model that combined tabular data, such as gender, age, and SUVmax, obtained with 3D DCNN is proposed. In addition, we investigated the diagnostic performance of a deep learning method based on 3D DCNN for discrimination of MPM from benign pleural disease using FDG-PET/CT imaging.

## RESULTS

MPM was diagnosed in 104 and benign pleural disease in 72 of the 176 patients in the test cohort (Table 1). Of the 104 MPM cases, cellular type was epithelial in 80, sarcomatoid in 11, biphasic in 9, and desmoplastic in 4. As for the 72 with benign disease, the diagnosis was benign asbestosis plaque, chronic fibrous pleuritis, benign pleural effusion, infectious (non-tuberculosis) pleuritis, chronic tuberculosis pleuritis/empyema, and active tuberculosis pleuritis in 31, 20, 14, 3, 3 and 1, respectively.

**Table 1:** Patient and tumor characteristics of three data sets

	Training data		Validation data		Test data	
	MPM	benign	MPM	benign	MPM	benign
Patients (*n*)	314	211	102	72	104	72
Sex						
Male/Female	259/55	190/21	84/18	64//8	85/19	69//3
Age						
Mean (y)	68.5 ± 9.2	70.5 ± 10.3	67.1 ± 8.3	71.8 ± 10.1	67.8 ± 9.9	70.3 ± 9.3
Range (y)	31–89	28–91	50–86	42–89	37–87	45–92
MPM						
Epithelial	237		77		80	
Sarcomatoid	41		13		11	
Biphasic	24		8		9	
Desmoplastic	12		4		4	
MPM stage						
I	76		26		26	
I	69		18		19	
III	83		29		30	
IV	86		29		29	
Benign pleural disease						
Benign asbestosic plaque		78		28		31
Chronic fibrous pleuritis		68		18		20
Benign pleural effusion		31		15		14
Infectious (non-tuberculosis) pleuritis		12		4		3
Chronic tuberculosis pleuritis/empyema		11		5		3
Active tuberculosis pleuritis		10		1		1
IgG4-related pleuritis		1		1		0
PET/CT machine						
Gemini GXL16	217	167	69	60	72	56
Gemini TF64	62	29	23	10	21	13
Ingenuity TF	15	8	2	0	4	2
Discovery IQ	20	7	8	2	7	1
SUVmax						
Mean	6.39 ± 4.90	1.11 ± 1.68	6.19 ± 4.55	1.19 ± 2.25	5.89 ± 3.96	0.98 ± 1.92
Range	0–46.8	0–9.08	0–21.3	0–12.25	0–15.3	0–11.84

Abbreviations: MPM: malignant pleural methothelioma; PET/CT: positron emission tomography/computed tomography; SUVmax: maximum standardized uptake value.

**Figure 1:**
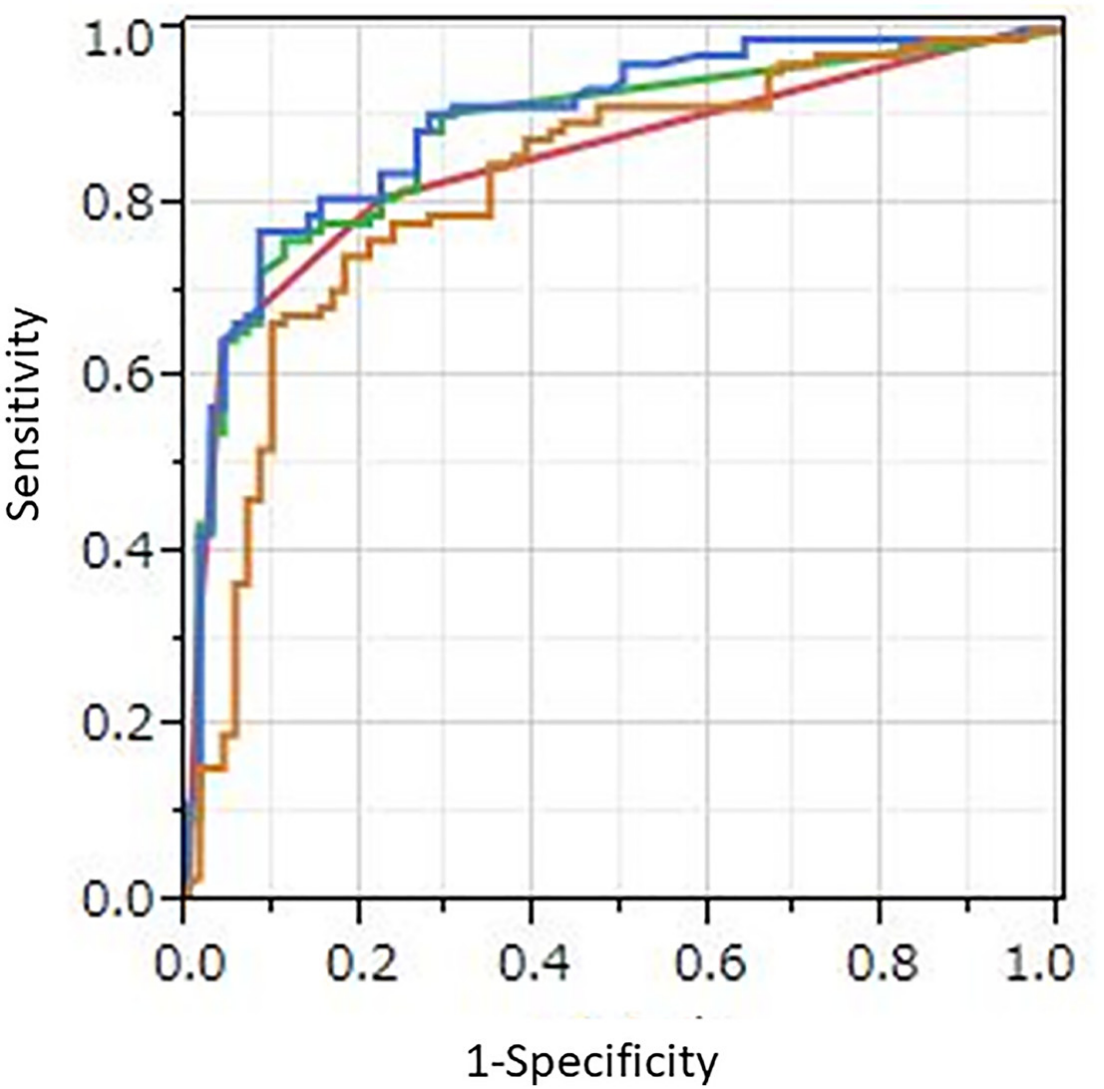
ROC curves for four protocols. Orange line: AI with PET/CT imaging alone (protocol A), red line: human visual reading (protocol B), green line: quantitative method using SUVmax (protocol C), blue line: AI with combined PET/CT imaging, SUVmax, gender, and age (protocol D). AUC values those protocols were 0.825, 0.854, 0.881, and 0.896, respectively. Protocol D showed significantly better diagnostic performance than protocol A, B, and C (*p* = 0.031, *p* = 0.0020, and *p* = 0.041, respectively).

Area under the curve (AUC) values obtained with receiver operating characteristic (ROC) analysis for protocols A, B, C, and D were 0.825, 0.854, 0.881, and 0.896, respectively (Figure 1, Table 2). As compared to A, B, and C, protocol D had significantly better diagnostic performance (*p* = 0.031, *p* = 0.0020, *p* = 0.041, respectively). A significant difference was also noted between protocols B and C (*p* = 0.026), whereas none between protocols A and B (*p* = 0.38), or between A and C (*p* = 0.086) was observed.

**Table 2:** Diagnostic performance of four protocols

	Sensitivity	Specificity	Accuracy	AUC
	95% CI	95% CI	95% CI	95% CI
AI with imaging	77.9% (81/104)	76.4% (55/72)	77.3% (136/176)	0.825
	69.9–85.9%	66.2–86.2%	71.1–83.5%	0.728–0.892
Human visual reading	80.8% (84/104)	77.8% (56/72)	79.5% (140/176)	0.854
	73.1–88.5%	68.2–87.4%	73.6–85.5%	0.775–0.908
Quantitative method using SUVmax	85.6% (89/104)	75.0% (54/72)	81.3% (143/176)	0.881
	78.8–92.3%	65.0–85.0%	75.5–87.0%	0.802-0.931
AI with combined imaging, SUVmax, sex, and age	88.5% (92/104)	73.6% (53/72)	82.4% (145/176)	0.896
	82.3–94.6%	63.4–83.8%	76.8–88.0%	0.818–0.943

Abbreviations: AUC: area under the receiver-operating-characteristic curves; CI: confident interval; AI: artificial intelligence; SUVmax: maximum standardized uptake value.

**Figure 2:**
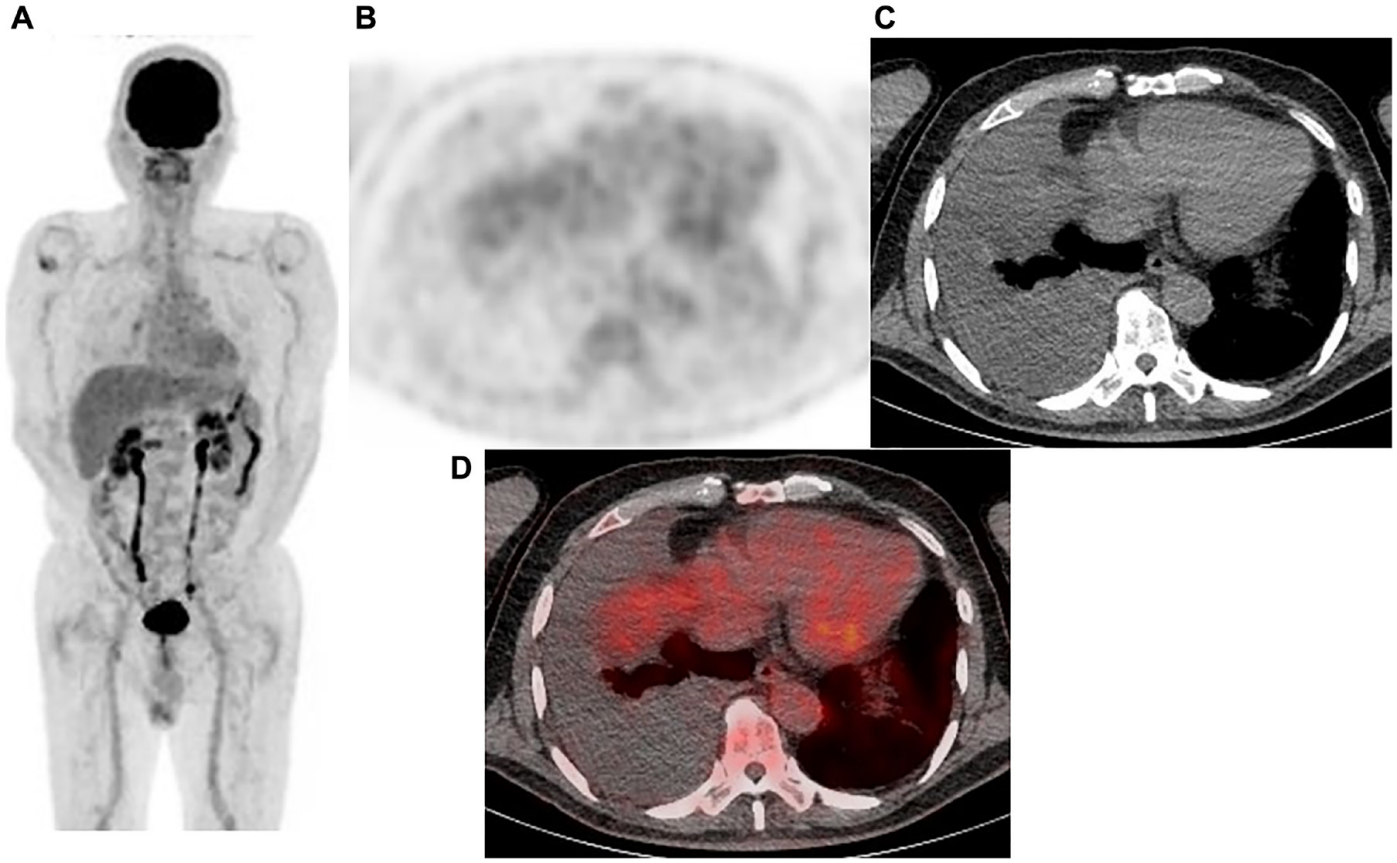
Representative case: 77-year-old male with right malignant pleural mesothelioma (epithelial type). (**A**) Maximum intensity projection (MIP) from FDG-PET, (**B**) Axial FDG-PET, (**C**) axial CT, and (**D**) fused FDG-PET/CT. Pleural effusion in the right pleural cavity was noted, while no FDG uptake was observed. The diagnosis was negative with protocol A (output value 0.127), B (grade 2), and C, whereas it was positive with protocol D (output value 0.448). VATS showed the sample to be positive, a malignant pleural mesothelioma (epithelial type).

**Figure 3:**
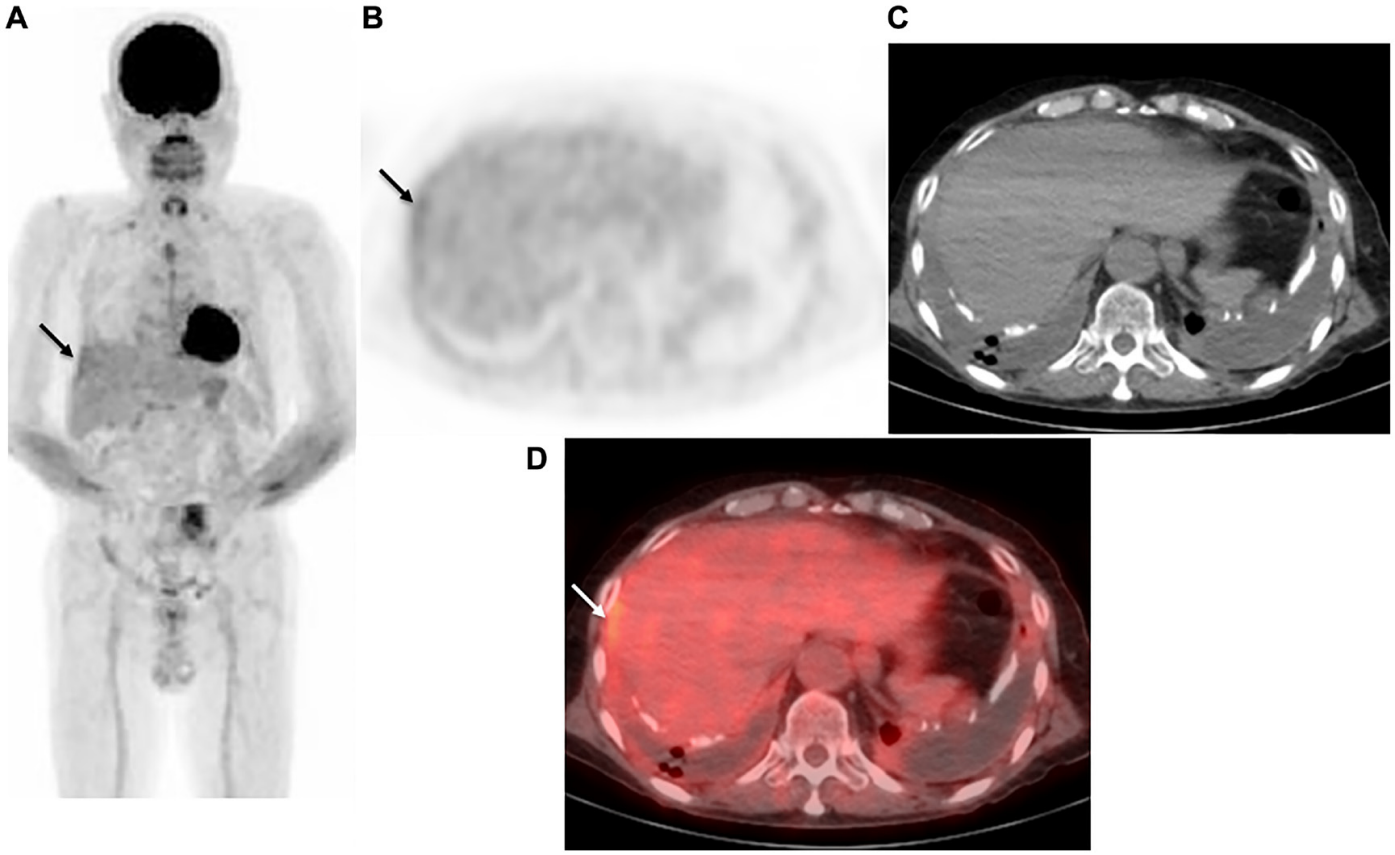
Representative case: 72-year-old male with right malignant pleural mesothelioma (epithelial type). (**A**) Maximum intensity projection (MIP) from FDG-PET, (**B**) axial FDG-PET, (**C**) axial CT, and (**D**) fused FDG-PET/CT. In the bilateral pleural cavity, pleural thickening, plaque, and calcification with effusion were seen, with weak FDG uptake (SUVmax 2.56) observed in the right thickened pleura (arrow). The case was diagnosed as negative with protocol B (grade 2). In contrast, protocol C and the AI models (protocol A with output value of 0.407, protocol D with output value of 0.649) indicated a positive diagnosis. VATS showed the sample to be positive, a malignant pleural mesothelioma (epithelial type).

To determine sensitivity, specificity, and accuracy of the visual reading analysis (protocol B), grades of 5, 4, and 3 were considered as positive, and grades of 2 and 1 as negative. Sensitivity, specificity, and accuracy for protocol B were 80.8% (84/104), 77.8% (56/72), and 79.5% (140/176), respectively (Table 2). As compared to benign pleural disease, the mean SUVmax value for MPM was significantly greater (5.89 ± 3.96 vs. 0.98 ± 1.92, *p* < 0.0001). To discriminate MPM from benign pleural disease, a best discriminative SUVmax cut-off of 2.23 was used based on ROC curve analysis, with sensitivity, specificity, and accuracy for protocol C of 85.6% (89/104), 75.0% (54/72), and 81.3% (143/176), respectively. For protocol A, we used a best discriminative output cut-off value of 0.333, and calculated sensitivity, specificity, and accuracy to be 77.9% (81/104), 76.4% (55/72), and 77.3% (136/176), respectively. As for protocol D, the best discriminative output value cut-off was determined to be 0.438, and those values were 88.5% (92/104), 73.6% (53/72), and 82.4% (145/176), respectively. The protocol D sensitivity was significantly higher than that of protocol A and B (*p* = 0.0026, *p* = 0.013, respectively), while protocol D accuracy was significantly higher as compared to protocol A (*p* = 0.0077). Findings obtained for two representative cases are shown in Figures 2 and 3.

## DISCUSSION

An AI subset is machine learning, in which a computer without specific programming is used to analyze relationships among existing data and then perform tasks based on the results [13, 14]. This is the first known study to examine the diagnostic performance of AI with a DCNN method and use of FDG-PET/CT images for discrimination of MPM from benign pleural disease. The results showed that AI with only FDG-PET/CT imaging was inferior to visual reading by a trained expert and a quantitative method using SUVmax. However, with the combination of PET/CT imaging, SUVmax, gender, and age, AI outperformed human visual reading and a quantitative method with SUVmax. Thus, a DCNN model combined with FDG-PET/CT imaging and clinical features was found capable of distinguishing between MPM and benign pleural disease, and demonstrated superior diagnostic performance.

Other recent studies have also investigated deep learning methods for diagnosis and prognosis prediction in patients with malignant tumors using FDG-PET/CT images, and reported their usefulness. Wang et al. [18] performed an evaluation of the diagnostic performance of FDG-PET/CT imaging for mediastinal lymph node metastasis in non-small lung cancer patients, which included pre-treatment results, and for human doctors found that sensitivity, specificity, and accuracy were 73%, 90%, and 82%, respectively, while those for a deep learning method were 84%, 88%, and 86%, respectively. In a study by Shen et al. [19], patients with definitive uterine cervical cancer and treated by chemoradiotherapy were enrolled, and the authors evaluated local relapse and distant metastasis predictions by deep learning using pre-treatment FDG-PET/CT results. For prediction of local relapse, they reported sensitivity, specificity, and accuracy values for deep learning of 71%, 93%, and 89%, respectively, while for distant metastasis those were 77%, 90%, and 87%, respectively. Furthermore, Pavic et al. [20] evaluated a radiomics model for predicting outcome in MPM patients using pre-treatment FDG-PET/CT images, and noted concordance index values for progression-free survival for their training and validation cohorts of 0.67 and 0.66, respectively.

The present study has several limitations. The retrospective design and use of cases treated at a single institution may limit generalization of the findings, while statistical errors may also be inevitable. It will be necessary to conduct a prospective multicenter study to validate the results. Furthermore, during the period of the study, a variety of scanners were used, and the reconstruction algorithm and acquisition parameters differed. Also, a single experienced reader made visual evaluations of the FDG-PET/CT images rather than a consensus approach with two readers. Finally, while various methods have been proposed to determine regions with the greatest impact for AI decision making [21], the present findings indicate that localization was not precise and the hints provided were not always correct. For collaboration between AI and clinician findings, additional methods should be considered and then validated with a larger dataset.

## MATERIALS AND METHODS

### Patients

Approval for this retrospective study was received from a local review board (No. 3456), which waived the requirement for patient-informed consent. Consecutive patients with histologically proven or suspected MPM based on history, physical examination, and chest CT findings (pleural thickening, fluid, plaque, calcification) underwent an FDG-PET/CT examination at our institution. As the examinations were performed on an intention-to-diagnose basis, all necessary procedures for obtaining a pathologic diagnosis were performed and cases of benign pleural disease were followed for at least three years. Histological diagnosis of MPM was determined based on results of a surgical biopsy performed during a VATS or thoracotomy examination, or CT-guided needle biopsy procedure. Benign pleural disease diagnosis was obtained from results of surgical biopsy specimens obtained during VATS or CT-guided needle biopsy, as well as results of cytologic evaluations of pleural fluid and needle aspiration pleural biopsy specimens, or of clinical and radiological follow-up examinations performed for at least three years. The pathologic examinations consisted of cytology, histology, and immunohistochemistry methods, depending on the diagnosis and diagnostic procedure. Patients with metastatic pleural disease or original malignant pleural disease other than MPM, as well as those who previously underwent a talc pleurodesis procedure were excluded from the present study.

The study cohort included 875 patients (751 males, 124 females; mean age 69.1 years; range 28-92 years) who underwent examinations from January 2007 to December 2017. MPM was diagnosed in 520 and benign pleural disease in 355 (Table 3). Cellular type for the 520 MPM cases was epithelial in 394, sarcomatoid in 65, biphasic in 41, and desmoplastic in 20. As for the 355 patients with benign disease, the diagnosis was benign asbestosis plaque in 137, chronic fibrous pleuritis in 106, benign pleural effusion in 60, infectious (non-tuberculosis) pleuritis in 19, chronic tuberculosis pleuritis/empyema in 19, active tuberculosis pleuritis in 12, and IgG4-related pleuritis in 2.

**Table 3:** Patient and tumor characteristics

Patients (*n*)	*N*	%
Number	875	
Sex		
Male/Female	751/124	85.8/14.2
Age		
Mean (y)	69.1 ± 9.6	
Range (y)	28–92	
Malignant pleural mesothelioma	520	59.4
Epithelial	394	45.0
Sarcomatoid	65	7.4
Biphasic	41	4.7
Desmoplastic	20	2.3
Stage of malignant pleural mesothelioma		
I	128	24.6
II	106	20.4
III	142	27.3
IV	144	27.7
Benign pleural disease	355	40.6
Benign asbestosic plaque	137	15.7
Chronic fibrous pleuritis	106	12.1
Benign pleural effusion	60	6.9
Infectious (non-tuberculosis) pleuritis	19	2.2
Chronic tuberculosis pleuritis / empyema	19	2.2
Active tuberculosis pleuritis	12	1.4
IgG4-related pleuritis	2	0.23

### FDG-PET/CT

Four different PET/CT scanners (Gemini GXL16, Gemini TF64, or Ingenuity TF, Philips Medical Systems, Eindhoven, The Netherlands; Discovery IQ, GE Healthcare, Waukesha, WI, USA) were available for FDG-PET/CT examinations during the time of the study. The patient was asked to fast for five hours prior to the scan. Blood glucose was determined immediately before injection of FDG at 4.0 MBq/kg body weight for the GXL16, 3.0 MBq/kg body weight for the TF64, or 3.7 MBq/kg body weight for the Ingenuity TF and Discovery IQ. All in the present cohort had a glucose level in blood lower than 160 mg/dL. Approximately 60 minutes after injection, static emission images were obtained. Helical CT scan imaging was performed from the top of the head to mid-thigh for attenuation correction and anatomic localization using the following parameters: tube voltage 120 kV (all four scanners), effective tube current auto-mA up to 120 mA (GXL16) 100 mA (TF64), 155 mA (Ingenuity TF) or 15~390 mA [Smart mA: noise Index: 25] (Discovery IQ), gantry rotation speed 0.5 seconds, detector configuration 16 × 1.5 mm (GXL16), 64 × 0.625 mm (TF64 and Ingenuity TF), or 16 × 1.25 mm (Discovery IQ), slice thickness 2 mm, and transverse field of view 600 mm (GXL16, TF64, Ingenuity TF) or 700 mm (Discovery IQ). Immediately following completion of CT scanning, PET imaging was performed from the head to mid-thigh for 90 (GXL16, TF64, Ingenuity TF) or 180 (Discovery IQ) seconds per bed position in three-dimensional mode, during which the patient was instructed to breathe normally. For reconstruction of attenuation-corrected PET images, a line-of-response row-action maximum likelihood algorithm was used for the GXL16, while for the TF64 and Ingenuity an ordered-subset expectation maximization (OSEM) iterative reconstruction algorithm (33 subsets, 3 iterations) was used, and Q.Clear block sequential regularized expectation maximization (BSREM) (β = 400) was used for the Discovery IQ.

### Data set

For analysis, FDG-PET/CT DICOM images were converted using the TFS-01 viewing software package (Toshiba Medical Systems, Tochigi, Japan) to JPEG format at 512 × 512 pixels. Patient number, gender, age, histopathology of MPM or benign pleural disease, PET/CT device, and harmonized lesion SUVmax from training, validation, and test data are presented in Table 1. Training and validation data were distributed at a ratio of 3:1 (i.e., 525:174 cases) and the remaining 176 cases were used for test data. Random numbering was used to randomly distribute the cases, with 314 MPM and 211 benign cases used as the training set, and 102 MPM and 72 benign cases as the validation set. For the test phase, 104 MPM and 72 benign cases were used. We compared four datasets, including AI with PET/CT imaging alone (protocol A), human visual reading (protocol B), a quantitative method using SUVmax (protocol C), and AI combined with PET/CT imaging, SUVmax, gender, and age (protocol D).

### Deep learning with DCNN

**Figure 4:**
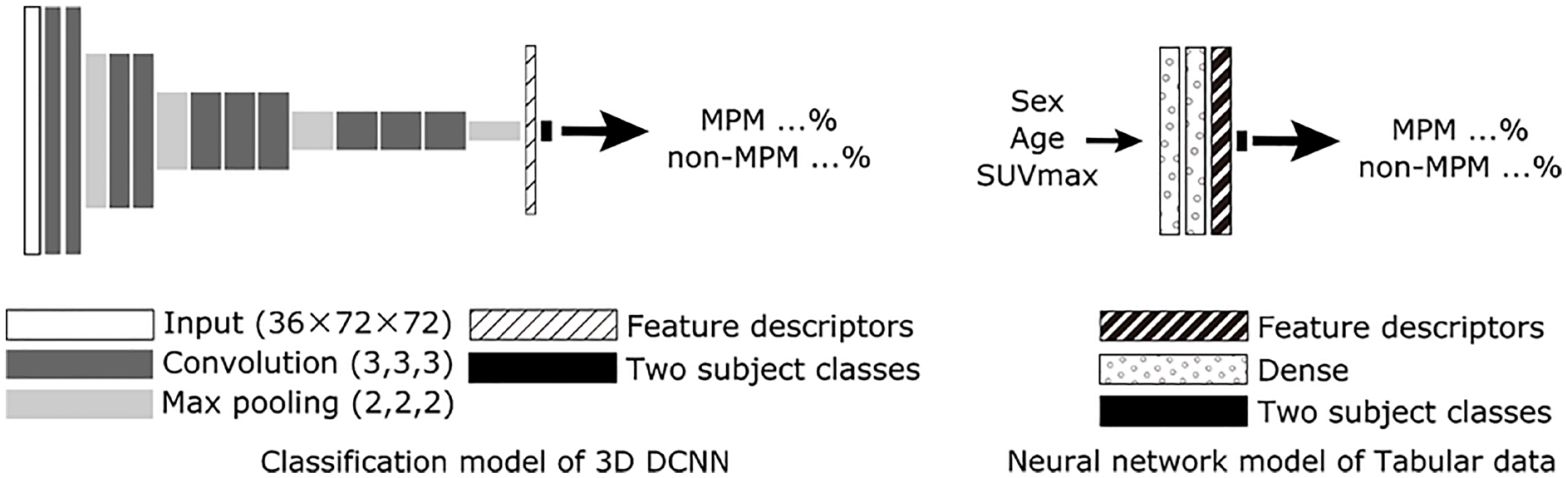
Scheme for proposed AI with PET/CT imaging alone (3D DCNN classification model, protocol A) and neural network model of table data. For the 3D DCNN, a 3D extended model based on VGG12 was utilized. A neural network model of tabular data was used to identify gender, age, and SUVMax in the combined network model. Abbreviations: DCNN: deep convolutional neural network; MPM: malignant pleural mesothelioma.

**Figure 5:**
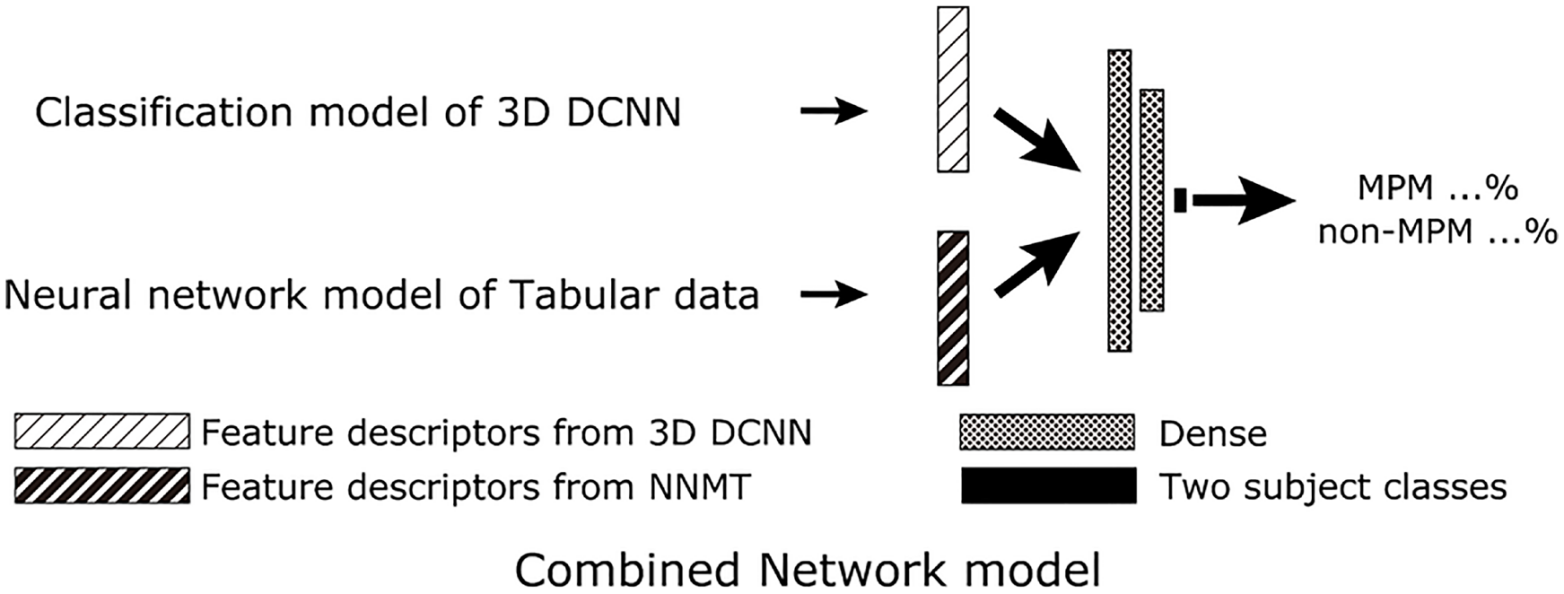
Scheme for proposed AI with combined PET/CT imaging, SUVmax, gender, and age (protocol D). For the combined network model, feature descriptors just before the final output layer of the classification model of 3D DCNN and the neural network model of tabular data were combined to create the final two class classifications of MPM and non-MPM. Abbreviations: DCNN: deep convolutional neural network; MPM: malignant pleural mesothelioma; NNMT: neural network model of tabular data.

A two-stage model was employed. The first stage was a classification model using a 3D DCNN and a neural network model of tabular data (NNMT), while for the second stage, a neural network was utilized to classify MPMs and non-MPMs based on feature descriptors (Figures 4 and 5).

A 3D extended version of the VGG12 network structure was used as a 3D DCNN classification model for the first stage, as suggested by the Visual Geometry Group at the University of Oxford in ILSVRC2014. After extracting the chest part, PET DICOM images were used as input data, then formatted as 3D data and resized to 36 × 72 × 72 pixels. Training of the 3D DCNN was done with 3D data from training data cases, with output results of the validation data used to indicate the end of training (protocol A). The NNMT was comprised of two dense layers, with tabular data (gender, age, SUVmax) used as input and two classes as output. Thereafter, training/validation data were used to train the NNMT.

The 3D DCNN and NNMT trained in the first stage were then used for the second stage. The 3D DCNN was inputted with 3D data and the NNMT with tabular data from the same cases, then feature descriptors were extracted just prior to the output layer for each network. The two feature descriptors were then inputted to a combined network model that consisted of two dense layers (protocol D) and training/validation data were used to train the model. Since both the 3D DCNN and combined network model provided output probabilities for MPM and non-MPM, the non-MPM probability was determined as the final score and used for subsequent comparisons. Therefore, output values for the 3D DCNN and combined network model were continuous values between 0 and 1.

All data processing was performed with a workstation (CPU: Core i7-9800X at 3.80 GHz, RAM 64 GB, GPU: TITAN RTX), with Python (version 3.6.8) (http://www.python.org) as the programming language and TensorFlow (version 2.2) (http://tensorflow.org/) as the deep learning framework. The optimizer used was Adam, with a learning rate of 1.0 × 10^–5^. Network training was performed with a batch size of 16 and up to 100 epochs, and was stopped when loss of the validation set was not improved. For each epoch, all training set structures were processed. After constructing the models, the accuracy of protocol A (AI with PET/CT imaging alone) and protocol D (AI with PET/CT imaging, SUVmax, gender, and age combined) was examined using the test image sets for the ability to distinguish MPM from benign pleural lesions.

### Radiologist review

A single board-certified nuclear medicine expert with 12 years of experience with oncologic FDG-PET/CT, and without knowledge regarding the clinical or histopathologic data of the cohort reviewed all FDG-PET/CT images in a retrospective manner. For assessment of MPM, diagnostic certainty was graded as 1 (definitely absent), 2 (probably absent), 3 (possibly present), 4 (probably present), or 5 (definitely present) based on visual analysis (protocol B). Additionally, semiquantitative analysis was performed using SUVmax, defined as maximum concentration in the target lesion (injected dose/body weight) (protocol C). The commercially available GI-PET software package (AZE Co., Ltd., Tokyo, Japan), developed to harmonize SUVs obtained with different PET/CT systems using phantom data [22], was used. To evaluate whether SUVmax differentiated MPM from benign pleural disease and identify the best cutoff value, ROC curve analysis was performed.

### Statistical analysis

To calculate the area under the ROC curve (AUC), analyses to determine the performance of the four protocols for distinguishing MPM from benign pleural disease were performed using the test data. Using Cochran’s *Q* test, the test data set was used to calculate sensitivity, specificity, and accuracy for differentiating MPM from benign pleural disease. Differences between any two protocols were tested using McNemar’s test with Bonferroni correction. Statistical analyses were performed using SAS, version 9.3 (SAS Institute Inc., Cary, NC, USA), with *p* < 0.05 considered to be significant.

## CONCLUSIONS

For differential diagnosis of MPM, 3D DCNN deep learning with the combination of FDG-PET/CT imaging and clinical features is a novel tool that is flexible and potentially useful. For assisting radiologists with diagnosis of MPM cases, the combined network model noted in the present study used with FDG-PET/CT is considered to be very helpful.

## Abbreviations

MPM: malignant pleural mesothelioma
CT: computed tomography
VATS: video-assisted thoracoscopic surgery
FDG: ^18^F-fluorodeoxyglucose
PET/CT: positron emission tomography/computed tomography
SUVmax: maximum standardized uptake value
ROC: receiver operating characteristic
DCNN: deep convolutional neural network
AI: artificial intelligence
2D: two-dimensional
3D: three-dimensional
OSEM: ordered-subset expectation maximization
BSREM: block sequential regularized expectation maximization
NNMT: neural network model of tabular data
